# Report of an Otic Capsule Disrupting Fracture of the Temporal Bone: Visualization of Pneumolabyrinth and Functional Assessment

**DOI:** 10.7759/cureus.12425

**Published:** 2021-01-02

**Authors:** Dávid Molnár, Marléne Mező, Zita Vaska, Zsuzsanna Sevecsek, Frigyes Helfferich

**Affiliations:** 1 Department of Otorhinolaryngology and Head and Neck Surgery, Medical Centre, Hungarian Defence Forces, Budapest, HUN; 2 Department of Anatomy, Histology and Embryology, Semmelweis University, Budapet, HUN

**Keywords:** temporal bone, trauma, three-dimensional imaging, pneumolabyrinth, semicircular canal, hearing loss

## Abstract

Blunt trauma of the head can result in temporal bone fracture. Different classifications exist, but from a functional perspective, to distinguish otic capsule sparing and otic capsule disrupting fractures is superior to the classic nomenclature. Disruption of the otic capsule is often associated with sensorineural hearing loss, vestibular dysfunction, cerebrospinal leakage, or even intracranial consequences. Pneumolabyrinth describes the condition when air is enclosed within the inner ear. It is a result of a pathological communication between the labyrinth and the middle ear spaces that often occurs due to trauma. It is not a ubiquitous but obvious radiographic hallmark of otic capsule violation. The present case is about a young woman who suffered a temporal bone fracture that involved the right lateral semicircular canal. Multiplanar and segmentation images were generated to depict the pneumolabyrinth developed in the lateral semicircular canal. Despite the preserved hearing, vestibular dysfunction was registered during the video head impulse test and videonystagmography. Treatment of pneumolabyrinth after temporal bone fracture can be a matter of surgery or conservative therapy. In the present case, we preferred conservative therapy because of the absence of serious consequences. Nevertheless, timing and the type of therapeutic modality must be personalized.

## Introduction

High impact blunt head traumas can be associated with fracture of the temporal bone. The conventional classification for such fractures based on the idea of how they relate to the anatomical axis of the petrous pyramid. Longitudinal, transverse, and mixed categories were defined, however, their clinical correlation is ambiguous [1]. The development of complications like either conductive hearing loss (CHL) or sensorineural hearing loss (SNHL), facial nerve injury, or cerebrospinal fluid (CSF) leakage is not consistent with a given type of fracture. Thus, a clinically more relevant classification was established according to the involvement of the otic capsule (OC): otic capsule sparing (OCS) and otic capsule disrupting (OCD) fractures. The latter one is more often associated with vertigo, SNHL, CSF leak, or intracranial (IC) consequences [2,3]. Mafee introduced the term pneumolabyrinth for the phenomenon when air is entrapped within the labyrinth [4]. It is seldom found after the trauma but can be an indirect marker of an OCD fracture. In the present work, we aim to give a pictorial review of the case of a young woman who suffered a hearing sparing OCD fracture on the right side associated with ipsilateral pneumolabyrinth.

## Case presentation

The subject of this study was a 20-year-old woman, whose medical records were analyzed retrospectively. Each step of handling data was carried out according to the principles of the Helsinki Declaration. The aforementioned young woman didn’t have any chronic illness. Besides smoking and occasional alcohol consumption, she had no history of other substance use.

In May 2020, she had a syncope in public and suffered blunt head trauma. After regaining consciousness, she was transferred to the local traumatology center. She underwent a head and cervical spine computed tomography and primary trauma assessment. Imaging didn't report any intracranial alterations, thus the patient was transferred to our emergency department where any life-threatening emergencies were ruled out. Due to the lack of prior CT images and constant dizziness, another cranial CT scan was ordered (with a slice thickness of 1.25 mm). The radiologist described a fracture of the right temporal bone next to the temporomandibular joint with small inclusions of air intracranially behind the pyramid and in between the soft tissues of the occipital muscles. Blood-like opacity filled the tympanic cavity. The ED initiated the patient’s admission to our otolaryngology department.

Upon arrival, the patient was unable to walk, complained about vertigo, and right-sided hearing loss. Hematotympanum was confirmed. Weber tuning fork test was lateralized to the right. Rinne test was negative on the right. Second-degree left beating horizonto-rotatory nystagmus was visible under Frenzel-goggles. Clinical head impulse test (cHIT) showed saccades when turning to the right. For supportive therapy, she received a combination of oral cinnarizine 20 mg and dimenhydrinate 40 mg twice a day. Oral amoxicillin + clavulanic acid was administered for a prophylactic purpose. SARS-CoV-2 RT-PCR was negative. From our primary assessment, we concluded the patient had a CHL and a putative vestibular dysfunction on the right side.

The radiology report of the fracture didn't explain the clinical symptoms. We examined the CT scans ourselves and identified a fracture involving the right lateral semicircular canal (SCC) and pneumolabyrinth next to its ampullary end. The mean value of -900.5 Hounsfield Units (HU) measured in the center of the bubble-like structures confirmed their air nature. We gave feedback to the radiology department, and to confirm our observations, they ordered a high-resolution CT (HR-CT) scan focused on the pyramid.

**Figure 1:**
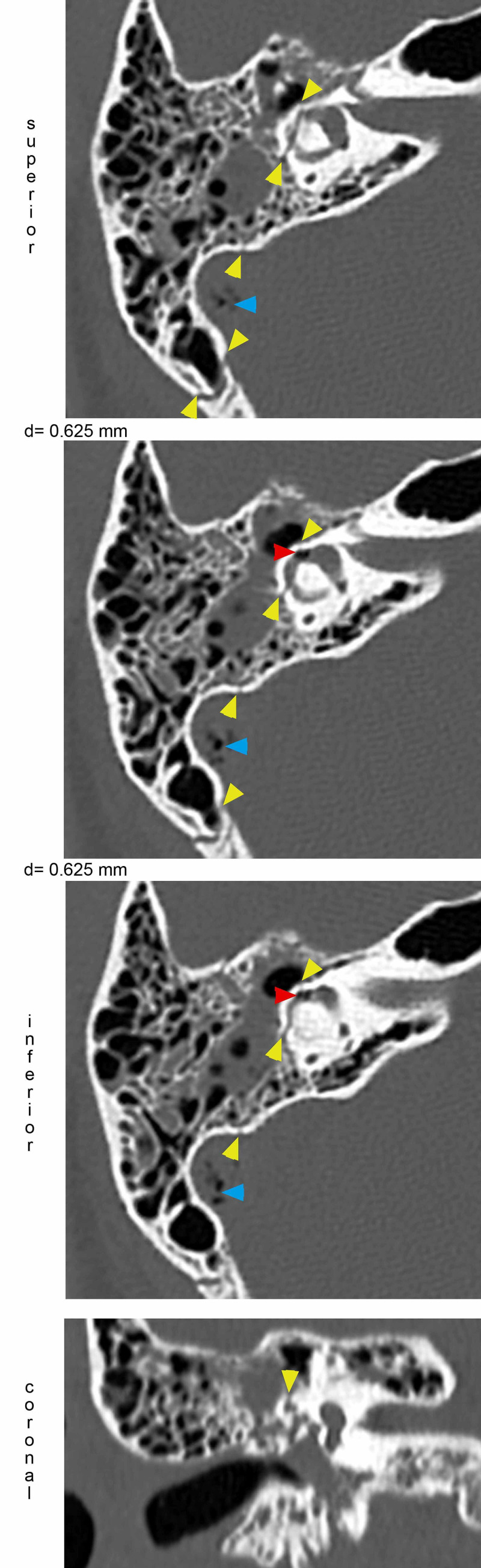
Demonstration of the fracture through the lateral SCC resulting in pneumolabyrinth. Axial CT images demonstrating pneumolabyrinth (red arrowheads) inside the right lateral SCC close to the ampulla. The fracture (yellow arrowheads) has a longitudinal trajectory disrupting the lateral SCC. Involvement of the lateral SCC is also visible in the coronal plane. Air bubbles are also present within the sigmoid sinus. Differences (d) of levels were 0.625 mm each. Section thickness: 0.625 mm. SCC: semicircular canal

**Figure 2:**
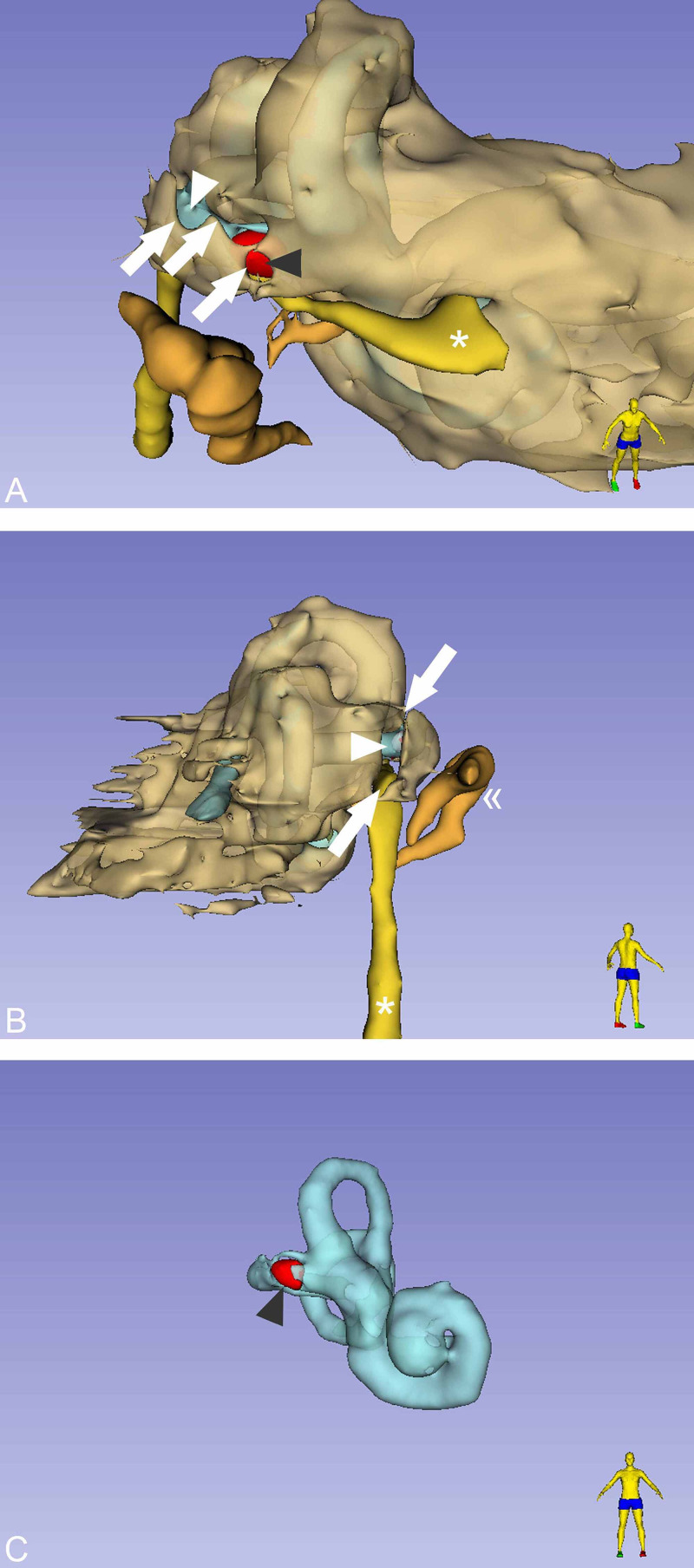
Three-dimensional volume rendering segmentation of the temporal bone. Postures of the homunculi stand for spatial orientation. (A) The red-colored area (grey arrowhead) demonstrates the localization of the entrapped air within the lateral SCC. The membranous labyrinth (white arrowhead) is visible through the fracture line (white arrows). Genu of the facial nerve (asterisk). (B) From a posterior view, the membranous labyrinth (white arrowhead) is visible again through the fracture line (white arrows). Ossicular chain (double angle quotation mark). Mastoid segment of the facial nerve (asterisk). (C) Segmentation of the membranous labyrinth and the pneumolabyrinth (gray arrowhead). SCC: semicircular canal

**Figure 3:**
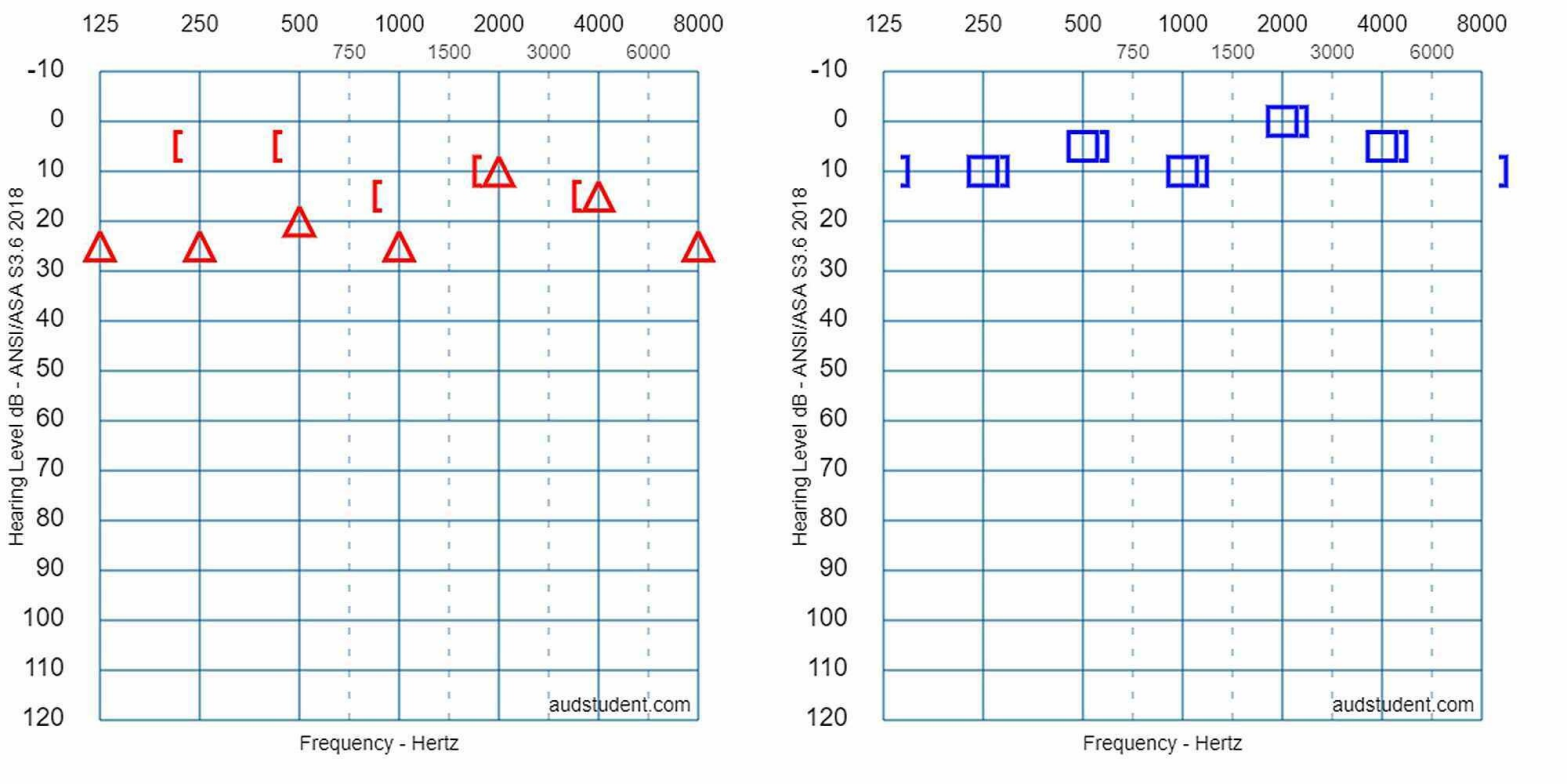
Initial pure tone audiometry curves. PTA showed a mild conductive hearing loss on the right side. Where the air-bone gap is present, its mean value is 15 dB. PTA: pure tone audiometry

**Figure 4:**
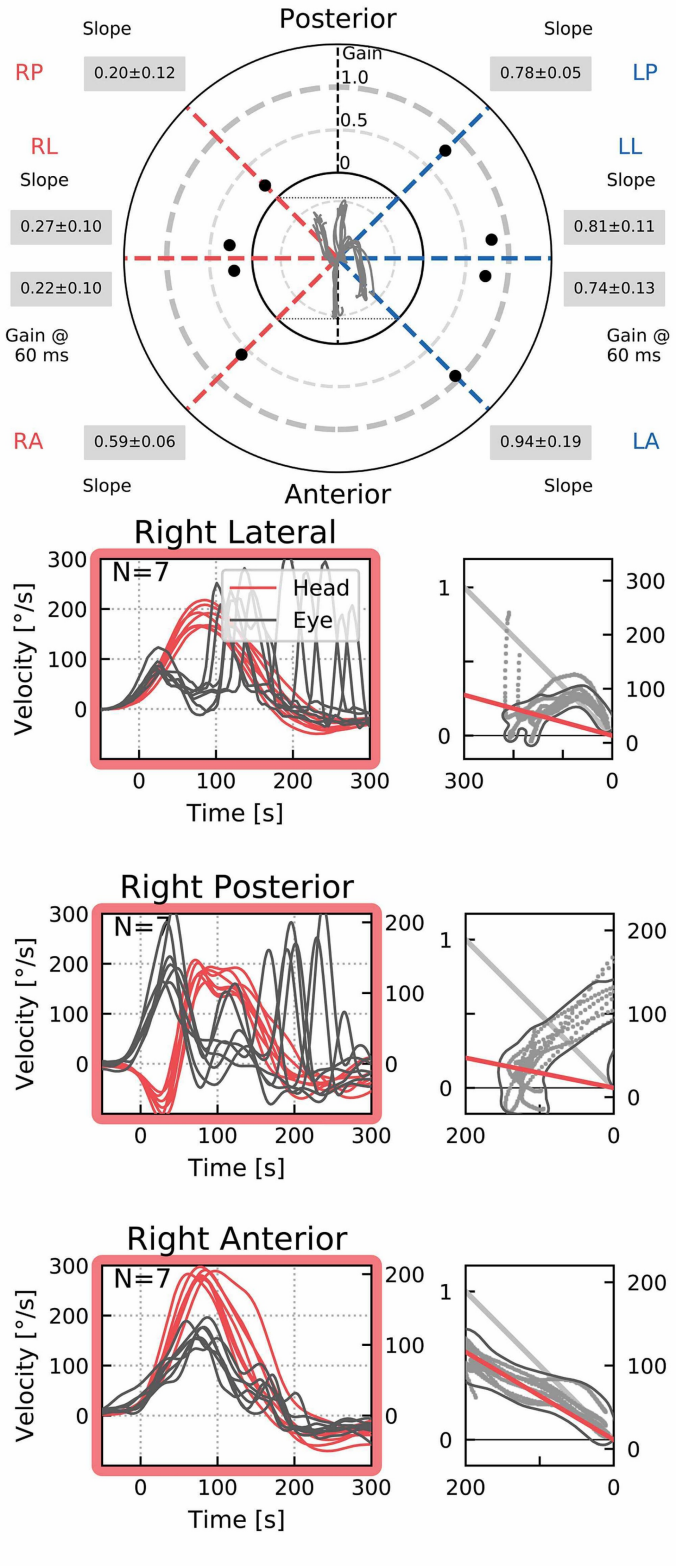
Video head impulse test report. vHIT confirmed impaired vestibulo-ocular reflex gain in all SCC planes on the right side. Graphs show covert and overt saccades in the corresponding SCCs. vHIT: video head impulse test; SCC: semicircular canal

**Figure 5:**
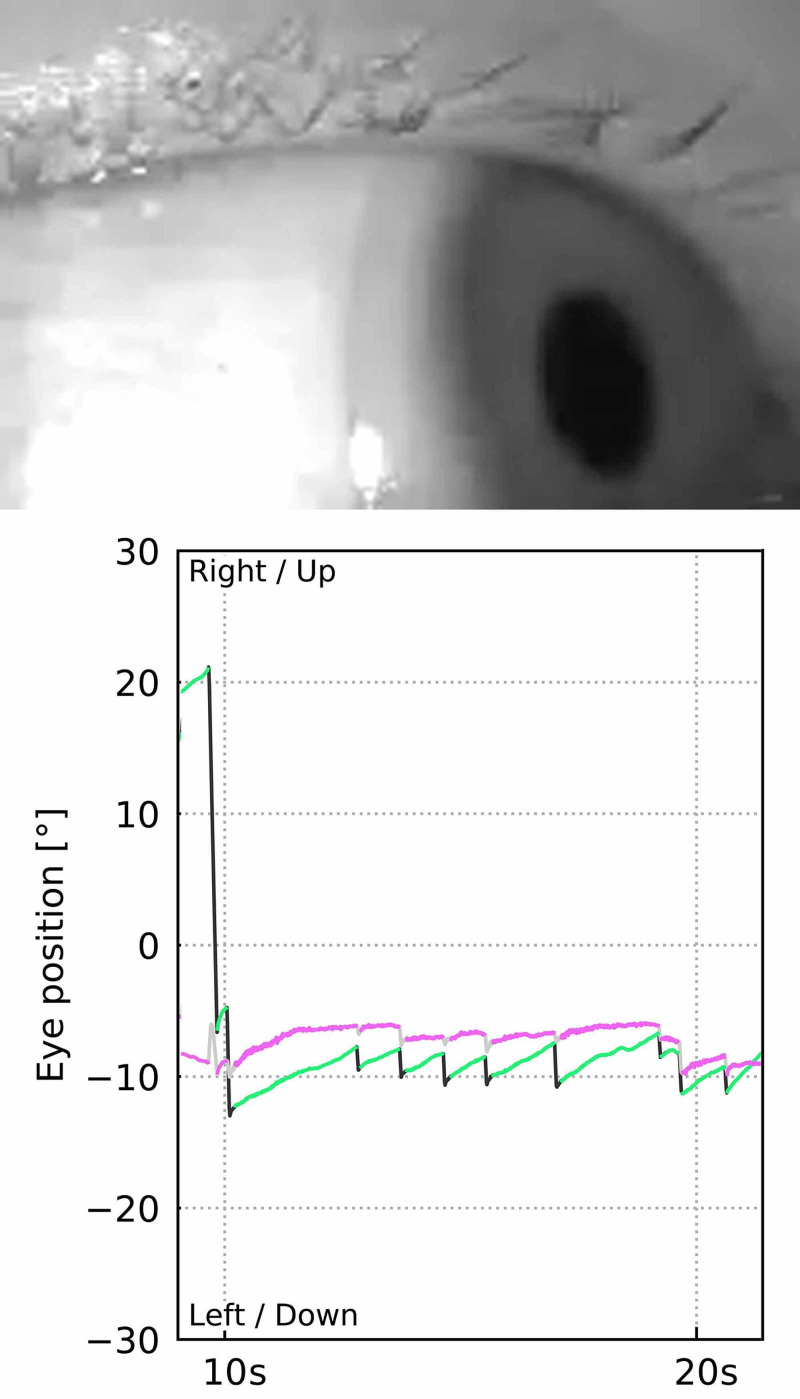
Videonystagmography pattern tracked with the vHIT goggles. A second-degree, left-beating horizonto-rotatory nystagmus was recorded. A demonstrative strip of eye-tracking shows the aforementioned nystagmus pattern when watching toward left side. vHIT: video head impulse test

The fracture involved the right lateral semicircular canal (Figure 1). Airy inclusions were visible within the sigmoid sinus, raising concern about the involvement of its dural wall. Negotiation with the neurosurgeon on-call didn't claim any intervention for it. In three-dimensional segmentation images (Figure 2) it is more spectacular how the pneumolabyrinth developed within the lateral SCC, where the fracture passes through the otic capsule. The hematotympanum explained the mild conductive hearing loss on the right side confirmed by pure tone audiometry (PTA) (Figure 3). To evaluate the vestibular dysfunction, a video head impulse test (vHIT) was carried out in all planes of the SCCs (Figure 4). As a response for high-velocity impulses high-amplitude overt and covert saccades were recorded during testing the right lateral and anterior semicircular canals. We registered small-amplitude covert saccades against stimuli of the right posterior canal. Vestibulo-ocular reflex (VOR) gains were significantly lower in all planes confirming a global impairment of the right vestibular apparatus. VOR gains were normal on the left. Eye-tracking confirmed a second-degree, left-beating, horizonto-rotatory nystagmus (Figure 5). Instrumentation for testing vestibular evoked myogenic potentials was not available (VEMP).

Since the patient didn’t develop severe SNHL or signs of IC complications, she was treated conservatively. Vertigo gradually decreased, and the patient was discharged six days after admission.

**Figure 6:**
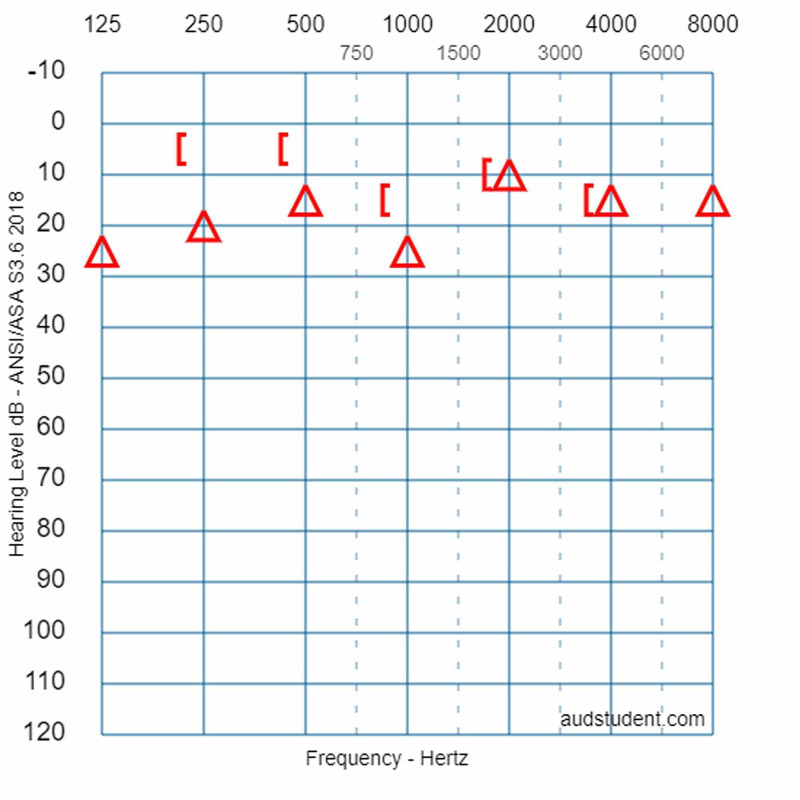
Follow-up pure tone audiometry. PTA was repeated one month after the previous assessment. The audiogram shows a narrowing air-bone gap on the right side. Hearing and the left side was identical to the previous measurement, therefore, it is not visualized here.

Fifteen days later she was scheduled for a follow-up. She complained about minor disequilibrium. cHIT showed saccades when turning to the right. Spontaneous nystagmus had resolved. The fistula sign was absent. Hematotympanum had cleared up. One month after the first PTA, a repeated examination showed narrowing of air-bone-gap (Figure 6). Since the first month of follow-up, the patient hasn’t appeared at scheduled visits.

Materials and methods

Multiplanar reconstructions and measurements were generated with RadiAnt DICOM Viewer v.4.6.9 (Medixant, Poznan, Poland). Volume rendering and 3D reconstruction were created with 3D Slicer v.4.10.2 [5]. Digital audiograms were prepared online with AudgenJS Beta 0.6 (https://www.audsim.com/audgenJS). vHIT and videonystagmography were recorded with the EyeSeeCam vHIT system (Interacoustics, Middelfart, Denmark).

## Discussion

The presence of air inside the labyrinth also called pneumolabyrinth, is a rare and abnormal finding which can be the result of communication between the middle ear spaces and the inner ear. The most common etiologies are trauma and iatrogenic manipulations around the oval window [4,6-10]. Traumatic pneumolabyrinth can occur in 7.1-48.4% of OCD fractures but is not present in the cases of OCS fractures, thus it can be an indirect marker of OC damage [7,8]. HR-CT is the modality of choice to detect pneumolabyrinth [6]. Since it is a rarity, CT scans can be misinterpreted. Some authors subdivide pneumolabyrinth into the cochlear, vestibule, or mixed forms [6,7,10]. Vestibular pneumolabyrinth is the most frequent [7]. The subject of the presented case suffered a rather longitudinal but OCD fracture of her right temporal bone which violated lateral SCC. Air was entrapped in the lateral SCC next to its ampullary end (Figures 1, 2). After multiple reports by emergency radiologists, final radiographic diagnosis and clinical evaluation were carried out by the otorhinolaryngology department. The mechanism of how air inclusions damage inner ear function is still unclear, however, the anatomical site may correlate with the type and severity of the symptoms. Experimental models showed that air inside the scala vestibuli resulted in the greatest impairment of hearing. Despite the remarkable fracture, the cochlear function was preserved and pneumolabyrinth was limited only to the affected SCC. Only a mild CHL was detected (Figure 3). It is questionable whether air inclusions themselves were the damaging factors or the trauma. It has been hypothesized that local inflammatory reactions and clotting may have a crucial role in the rapid sealing of the defect [11]. This can explain why the disruption of inner ear fluid spaces resulted in only vestibular damage (Figures 3, 4).

Treatment of traumatic pneumolabyrinth must be personalized: the patient’s history (e.g., prior ear surgery, co-morbidities), radio-morphologic features (site of the fracture, stapes displacement, anatomical variations, etc.), functional impairment (severity of hearing loss and disequilibrium) must be taken into consideration when deciding between explorative tympanotomy or conservative management [6,7,10,11]. In selected cases, surgical treatment can be rewarding even weeks after the onset of pneumolabyrinth [6,11]. In our present case, the absence of major complications (e.g., complete facial nerve paralysis or evidence of CSF leakage) and the gradual establishment of vestibular compensation convinced us to choose conservative treatment in the acute phase [12]. Elective subtotal petrosectomy (SP) has its rationale to prevent otogenic meningitis [12,13]. We informed the patient about a possible indication of such surgery in the future, depending on her clinical course. Unfortunately, the patient didn’t return for more follow-ups.

## Conclusions

Pneumolabyrinth is a rare condition and is often a result of traumatic injuries. Identification requires experienced eyes especially in a large patient volume ED. Radiologists should keep in mind, that otic capsule disruption can occur solely at the site of a single semicircular canal. Therapy can be either conservative or surgical but must be individualized. An increasing number of reported cases may improve our understanding of the pathophysiology of pneumolabyrinths and may help us defining appropriate therapeutic algorithms.
